# HIV-1 Diversity, Drug-Resistant Mutations, and Viral Evolution among High-Risk Individuals in Phase II HIV Vaccine Trial Sites in Southern China

**DOI:** 10.1371/journal.pone.0068656

**Published:** 2013-07-15

**Authors:** Haiyan Qi, Ke Zhao, Fei Xu, Xuzhao Zhang, Zhiyong Zhang, Li Yang, Chunling Li, Xu Liang, Weigui Guo, Shihai Chen, Zhihao Liu, Wenyan Zhang, Xiao-Fang Yu

**Affiliations:** 1 Cancer Institute, The Second Affiliated Hospital, Zhejiang University School of Medicine, Hangzhou, Zhejiang, China; 2 Institute of Virology and AIDS Research, First Hospital of Jilin University, Changchun, Jilin, China; 3 School of Public Health, Guangxi Medical University, Nanning, Guangxi, China; 4 Centers for Disease Control and Prevention, Baise, Guangxi, China; 5 Centers for Disease Control and Prevention, Beihai, Guangxi, China; 6 Centers for Disease Control and Prevention, Nanning, Guangxi, China; 7 Department of Molecular Microbiology and Immunology, Johns Hopkins Bloomberg School of Public Health, Baltimore, Maryland, United States of America; Institut Pasteur of Shanghai, Chinese Academy of Sciences, China

## Abstract

HIV-1 prevalence in Guangxi, China, has been growing since 1996, when the first case was reported. Over half of HIV-1 positive patients in Guangxi Province were injecting drug users (IDUs), possibly because of the province’s location near drug-trafficking routes. Since a phase II HIV vaccine trial is ongoing there, a current characterization of the subtypes of HIV-1 among IDUs in Guangxi would provide critical information for future HIV vaccine trials, as well as further control and prevention of HIV-1 transmission. Thus, we conducted a molecular epidemiological investigation of HIV-1 samples from 2008–2010 among IDUs in multiple cities in Guangxi Province. Our results, based on the *gag/pol* fragment, indicated a very high proportion (78.47%) of HIV-1 CRF08_BC recombinants, some CRF01_AE (15.38%) recombinants, and a low proportion of CRF07_BC (6.15%) recombinants among the IDUs. The high proportion of CRF08 HIV-1 strains among recent IDUs matches the vaccine candidate constructs. However, future vaccine development should also incorporate CRF01-targeted vaccine candidates. Distinct Env sequence evolution patterns were observed for CRF08_BC and CRF01_AE, indicating that different local selection pressures have been exerted on these two HIV-1 subtypes. Unique drug-resistant mutations were also detected, and our data indicate that HIV treatment programs should consider pre-existing drug-resistant mutations.

## Introduction

Human immunodefiency virus type 1 (HIV-1) infection has continued to be a global epidemic problem since its discovery in the early 1980s [1]. HIV-1 prevalence in China has grown in recent years [2]. Guangxi Province experienced one of earliest and heaviest epidemics of HIV in China [3]–[5]. Two major circulating HIV-1 strains, CRF08 BC and CRF01 AE, have been reported in Guangxi and have been selected for HIV-1 vaccine development [6]–[8]. Guangxi is also the site of an ongoing phase II HIV vaccine trial.

HIV infection in Guangxi has been increasing dramatically since the first reported case in 1996 [9]. Evidence indicates that the transmission of HIV-1 followed the heroin traffic routes from Yunnan Province in the west and Vietnam in the south [3], [7]. Several reports have suggested that different genotypes of HIV-1 have predominated in different cities within this one province: for example, CRF08_BC in Baise (western Guangxi) and Nanning (central Guangxi) and CRF01_AE in Pingxiang (southeastern Guangxi) [10]–[12]. However, the current evolution status of HIV-1 in Guangxi Province is not clear. Because of the wide use of anti-HIV therapy, more and more strains with drug-resistant mutations are being selected and transmitted, even among treatment-naïve HIV-positive patients [1], [10], [12]. Currently, anti-HIV drugs are divided into three categories: Protease inhibitors (PIs) target HIV-1 protease, whereas both nucleoside analog reverse transcriptase inhibitors (NRTIs) and non-nucleoside reverse transcriptase inhibitors (NNRTIs) target the HIV-1 reverse transcriptase. Thus far, dozens of amino acid residues of HIV-1 have been confirmed to be selectively mutated by antiviral drugs, and mutations in these residues give rise to HIV-1 strains resistant to certain drug(s). Thus, screening these residues could provide critical information for antiviral treatment.

In the present study, we have performed phylogenetic analyses and detailed sequence analyses of *gag*, *pol*, and *env* sequences from 65 HIV-1-infected injection drug users (IDUs) in Baise, Beihai, and Nanning cities of Guangxi Province, and we present evolutionary information based on both the *gag/pol* and *env* genes. These new findings have important implications for vaccine development and evaluation. The results of our screening for possible mutations among these *gag/pol* sequences also provide information on the current status of circulating drug-resistant HIV-1 strains and suggest ways to further improve antiviral treatment within the area.

## Materials and Methods

### Ethics Statement

Written consent was obtained from all participants involved in the study. This study was reviewed and approved by the institutional review board of Guangxi Medical University in Nanning, Guangxi, China.

### Study Subjects

HIV-positive subjects from the cities of Beihai, Baise, and Nanning in Guangxi Province, China, were identified through annual surveillance by local Centers for Disease Control among 705 IDUs between 2008 and 2010. In total, 65 HIV-1 positive patients were selected for this study on the basis of the following two criteria: being an IDU and being anti-HIV-1 treatment-naïve. After pretest counseling, informed consent was obtained from each enrolled participant. Questionnaires completed by the participants included demographic and behavioral information. Blood samples were collected from IDUs for serologic testing and were stored at −70°C.

### HIV Testing

Serum samples retrieved from IDUs were tested for HIV-1 antibody by enzyme-linked immunosorbent assay (ELISA; Organon Teknika, Boxtel, The Netherlands). All ELISA-positive samples were analyzed with an HIV-1/2 Western blot immune assay (Gene Laboratory, Singapore). Samples positive for both tests were subjected to the experiments described below.

### HIV-1 Subtyping by Polymerase Chain Reaction (PCR)

DNA samples corresponding to 1×10^5^ uncultured peripheral blood mononuclear cells (PBMCs) from HIV-1-positive individuals were retrieved with a DNeasy Blood & Tissue Kit (QIAGEN, CA) according to the manufacturer’s protocol and subjected to PCR. The primers for *gag/pol* GAG-POL-F1, 5′-GTCCAAAATGCRAAYCCAGA-3′ (nt 1756-1775) and GAG-POL-R1 5′-TGGAGYTCATAHCCCATCCA-3′ (nt 3234-3253), or *env* C2V5-F1, 5′-CTCCAGCTGGTTWTGCRATT-3′ (nt 6880-6899) and C2V5-R1 5′-GCCTGTACCGTCAGCGTTAT-3′ (nt 7827-7846), were used in separate reactions under the same conditions: 95°C for 5 min, 25 cycles at 94°C for 30 sec, 55°C for 30 sec, and 72°C for 1.5 min. A sample (5 µl) of the first-round PCR product was used in the second-round reaction, which consisted of a 50-µL reaction mixture containing 2.5 mM of deoxyribonucleotide triphosphate, 10×PCR buffer (Mg^2+^ Plus, containing 15mM Mg^2+^), 5 U/µl of Taq DNA polymerase (TaKaRa, DR100A), and 20 µM of the inter-nested primers for GAG-POL-F2 5′-ACAGCATGTCAGGGAGTGG-3′ (nt 1831-1849) and GAG-POL-R2 5′-ATTGCTGGTGATCCTTTCCA-3′ (nt 3006-3025) for the *gag/pol* region, or C2-V5-F2(inter) 5′-CAGCTGGTTWTGCGATTCTAA-3′ (nt 6883-6903) and C2-V5-R2(inter) 5′-RTYYCCTCCTCCAGGTCTGA-3′ (nt 7627-7646) for the *env* product. Cycling conditions were 95°C for 5 min, followed by 35 cycles at 94°C for 30 sec, 61.6°C (annealing temperature for GAG-POL-F2/R2 primers) or 64°C (annealing temperature for C2-V5-F2/R2 primers) for 30 sec, and 72°C for 1.5 min.

### DNA Purification and Sequencing

PCR products were purified for sequencing by using the QIAquick PCR purification kit (Qiagen) according to the manufacturer’s protocols. Sequencing of PCR products was performed by Sangon Biotech Co., Ltd. (Shanghai, China) with an automated sequencer (PRISM automated sequencer, version 3100; ABI, Foster City, CA), following standard protocol suggested by the manufacturer. For an internal control, a random selection of 25% of the samples was re-amplified, sequenced, and analyzed. The sequences retrieved from the control group were identical to the previous sequencing results.

### Alignment and Phylogenetic Analysis

DNA alignment was performed by the ClustalW method under MEGA5 [13], followed by manual adjustment. Reference sequences were retrieved from the Los Alamos National Laboratory HIV Sequence Database (http://www.hiv.lanl.gov). Phylogenetic analysis was also conducted with MEGA5. Neighbor-joining trees were constructed using a Kimura 2-parameter model and tested by the bootstrap method for 1,000 replicates. Bootscan analysis was also performed with Simplot 3.5.1 [14] to detect possible recombination.

### Drug Resistance Analysis

All Gag/Pol sequences were submitted to the HIV Drug Resistance Database at Stanford University (http://hivdb.stanford.edu/) to detect possible potency against anti-HIV drugs. The *gag/pol* sequence generated above were used for the analysis because proviral DNA sequences may be the best source of drug-resistant mutations in this study, especially since all patients in this study were treatment-naïve; thus, there was no selection pressure exerted on the viral RNA by any antiviral drug. Also, assay targeting plasma RNA as representative of viral population may not detect drug resistant mutations in HIV-1 resoreviore (latantly infected cells), or minor virus variants or variants archived in PBMC DNA [15], [16]. These viral sequences can only be detected using DNA samples.

## Results

### Epidemiological Information for Selected IDU Patients

In total, 65 HIV-1 positive patients were selected from Beihai, Baise, and Nanning cities from Guangxi province on the basis of the following two criteria: being an IDU and being anti-HIV-1 treatment-naïve. The epidemiology information for these patients is presented in Table 1. Apparently, HIV-1-positive IDU patients from Beihai were relatively younger in age average age, 31.6 years old), while those from Baise and Nanning were older, with similar, mean ages of 38.1 and 36.9, respectively. Also, most IDU patients from Beihai were male; Baise and Nanning had more female IDUs. Unfortunately, in addition to the 47 adults, there were 18 teenagers who were determined to be HIV-1-positive, raising alarms concerning both drug control and HIV-1 prevention.

**Table 1:** Epidemiologic information concerning HIV-1-positive IDU from Guangxi, China.

City	Mean age (±S.D.)	Male/Female	Age on first drug use <25/>25
Baise	38.14 (±5.29)	14/8	12/10
Beihai	31.56 (±6.63)	22/1	21/2
Nanning	36.95 (±6.00)	15/5	12/8

### Phylogenetic Analysis of the *gag* and *pol* Regions

**Figure 1:**
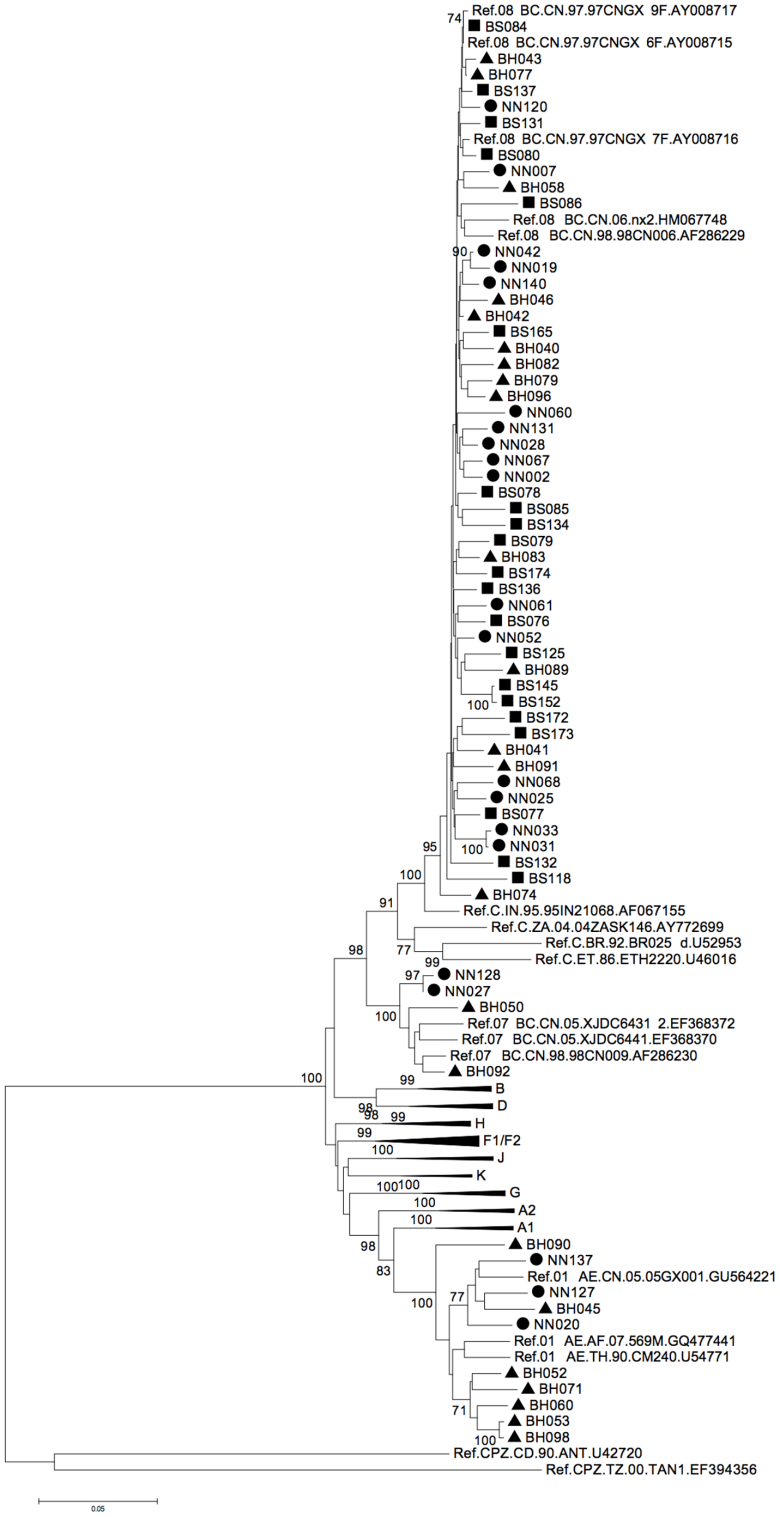
Phylogenetic analysis of HIV-1 sequences from Guangxi Province. The results indicate that various subtypes of HIV-1 were transmitted among the IDUs in Nanning and Beihai cities, while only the CRF08_BC subgroup was found in IDUs in Baise city. A total of 65 *gag/pol* sequences were examined, covering positions corresponding to 1850-2961 of the HXB2 genome. Circles indicate sequences from Nanning, triangles from Beihai, and squares from Baise city. Only bootstrap values >70% are shown.

Partial *gag/pol* gene sequences were successfully retrieved from all the selected antiviral-naïve HIV-1-positive IDU patients. Phylogenetic analyses (Figure 1) showed various subtypes among the 23 samples from Beihai city, including 14 CRF08_BC (60.87%), 7 CRF01_AE (30.43%), and 2 CRF07_BC (8.70%). Of the 21 sequences in Nanning, 16 (76.19%) were determined to be CRF08_BC (the local dominant subtype), while 3 (14.28%) were CRF01_AE and the remaining 2 (9.52%) CRF07_BC. In contrast, all 21 samples from Baise were classified as CRF08_BC, showing a complete dominance within the area.

To determine whether the newly identified CRF07_BC and CRF08_BC contain the same breakpoints as those originally reported for CRF07_BC and CRF08_BC in the *gag/pol* fragment, a detailed bootscanning [17] was carried out using SimPlot 3.5.1, with a window size of 300bp and a step of 20bp [14]. The recombination breakpoints of our new CRF07_BC and CRF08_BC viruses were consistent with previous reports of CRF07_BC and CRF08_BC [6]–[8], [18].

**Figure 2:**
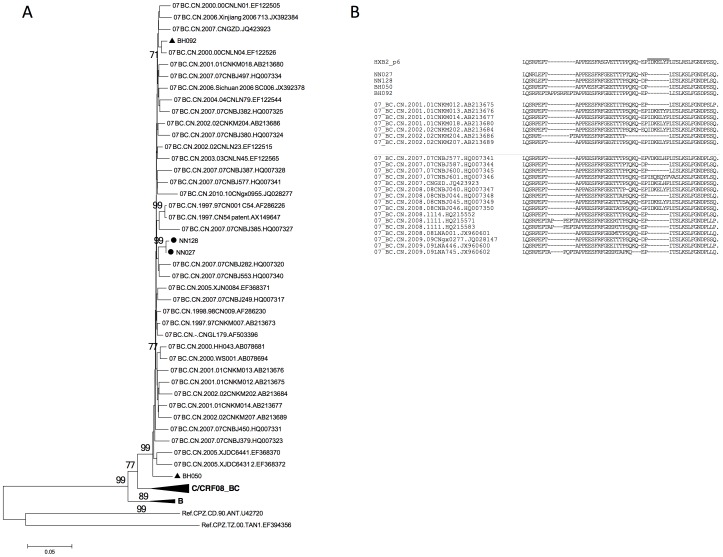
CRF07_BC sequences from Guangxi are not derived from Yunnan province. A. Phylogenetic analysis comparing new CRF07_BC sequences from Guangxi to other reported sequences from China has suggested that Guangxi CRF07_BC does not have a direct relationship to those from Yunnan province. *gag/pol* sequences corresponding to 1850-2961 of HXB2 were used for phylogenetic tree construction. B. A 7-aa deletion within the CRF07_BC P6 region was conserved among our Guangxi CRF07_BC sequences and could be easily detected within sequences from China, but was barely detectable in those from Yunnan. The position of the 7-aa deletion (IDKELYP) is indicated by the short line above the sequences.

CRF07_BC Gag sequences have rarely been seen in Guangxi province [12], [18]. To determine the possible origin of Guangxi CRF07_BC, another phylogenetic analysis was done with other sequences from China. As shown in Figure 2A, the Guangxi CRF07_BC strains most likely originated from cities in northern China, such as Xinjiang, Beijing, and Liaoning, but not from Yunnan, where CRF07_BC is also predominant [8]. Also, CRF07_BC sequences may contain a unique in-frame deletion in the p6 domain that is absent from the CRF08_BC and CRF01_AE sequences. Among all four *gag/pol* sequences retrieved from CRF07_BC strains, a 7-aa deletion (IDKELYP, as in the HXB2 sequence) could be detected in HIV-1 samples from both Beihai and Nanning cities (Figure 2B). Similar mutations have been reported for CRF07_BC Gag sequences identified from other regions in China (Figure 2B, and Ref [19]), but rarely in Yunnan province (Figure 2B), again suggesting that Yunnan may not be the origin of Guangxi CRF07_BC.

### Phylogenetic Analysis of Env Sequences

**Figure 3:**
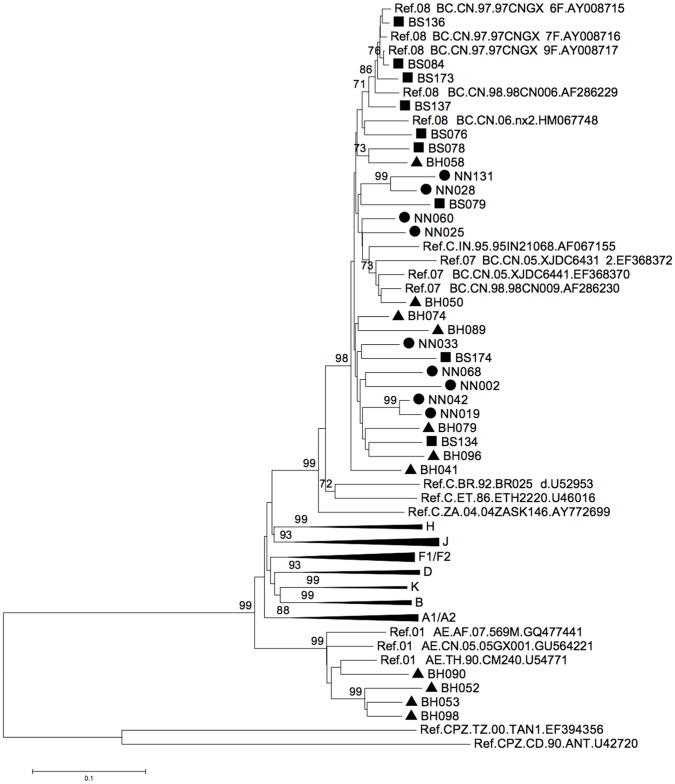
Phylogenetic analysis of partial *env* genes from Guangxi HIV sequences. A subtype classification similar to that for *gag/pol* was detected for each *env* gene. A total of 29 *env* sequences were included, covering positions corresponding to 6883-7642 of the HXB2 genome. Circles indicate sequences from Nanning, triangles from Beihai, and squares from Baise city. Only bootstrap values >70% are shown.

Partial *env* genes of 29 *gag/pol*-positive samples were retrieved and subjected to phylogenetic analysis (Figure 3); this analysis suggested a classification of these 29 sequences into a scheme similar to a previous one based on *gag/pol*; however, only the CRF01_AE sequences could be definitely determined, and not for those of the CRF07_BC or CRF08_BC sequences, since both the CRF07_BC and CRF08_BC sequences had a subtype C origin for this *env* fragment.

### Amino Acid Analysis of the C2-V4 Fragment of Envelope

**Figure 4:**
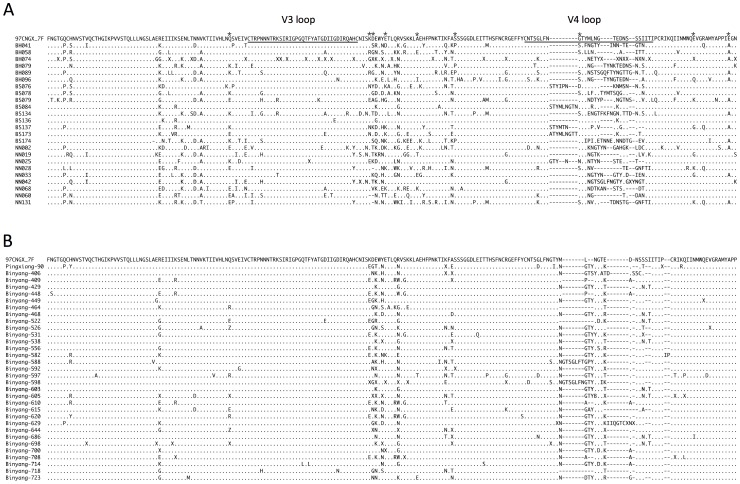
Sequence alignment of partial Env fragments (C2-V4) from Guangxi HIV-1 CRF08_BC sequences. Only subtype C-originating sequences from this study (A) and from the year 2000 (B) are included, with 97CNGX_7F as the reference sequence. The symbol “.” indicates identical amino acids when compared to 97CNGX_7F, “–” indicates gaps, and “X” indicates amino acids that cannot be determined. V3 and V4 loops are underlined. Residues associated with the Indian subtype C are labeled with an asterisk (*).

The alignment of all the CRF08_BC sequences (determined by the *gag/pol* sequences, Figure 4A) revealed a highly conserved V3 loop and a highly variable V4 loop in the partial Env fragment, which is consistent with previous studies [10], [20]. A conserved dodecapeptide, RIGPGQTFYATG [21], was found in 20 of 24 BC sequences, with the four exceptions being BS079, NN002, BH074, and NN025; the last two sequences contained an undetermined amino acid “X” and may in fact be identical to the 20 sequences. No similar fragment was observed in the V4 loops, which varied in both their sequence and residue length.

However, when we compared these sequences to the protein alignments of the same fragment in 32 samples retrieved in the very same province in 2000 [10] (Figure 4B), we observed a much higher degree of variation. To determine the evolutionary difference between CRF08_BC sequences obtained from Guangxi from 2008–2010 and those from the same area in 2000, we calculated the pairwise amino acid p-distance within these two groups using MEGA5. As a result, we found that the year 2000 sequences had an average p-distance of 0.058 (standard deviation [S.D.], 0.020; range, 0.000–0.117), while sequences from 2008–2010 had an average p-distance of 0.164 (S.D, 0.041; range, 0.025–0.240). The difference between the two groups was highly significant (p<0.001, Student’s test, two-tailed, with Microsoft Excel) in terms of the evolution of the sequences, meaning that recent Guangxi CRF08_BC strains were more evolutionarily different from each other, suggesting perhaps that these strains undergo adaption that allows them to infect people in this locality.

**Figure 5:**
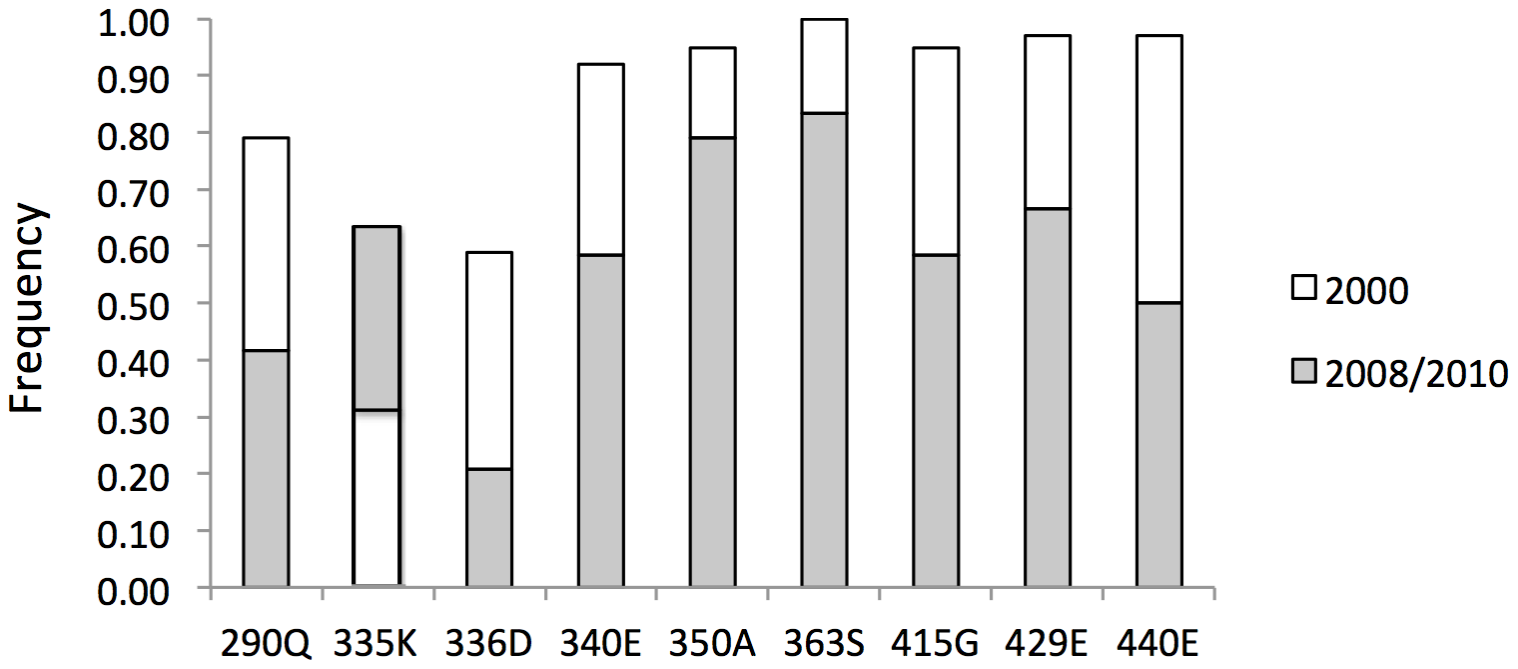
Frequency of identical amino acids detected in Env fragments (C2-V4) at positions associated with the India type C strain. The results indicate that samples from this study share fewer common residues than do samples from just 8–10 years ago, suggesting that an evolution of these sequences has been occurring for years. Positions are numbered according to the Env protein sequence of HXB2.

The CRF08_BC envelope sequences from Guangxi were previously determined to be of Indian origin [10]. However, different evolutionary patterns, with a generally lower residue frequency, were observed for these new Env fragments at the following residues associated with Indian type C (given here are the HXB2 location and amino acid residue, frequency; these positions are marked in Figure 4A with asterisks): 290Q, 0.42; 335K, 0.63; 336D, 0.21; 340E, 0.58; 350A, 363S, 0.83; 415G, 0.58; 429E, 0.67; 440E, 0.50, as compared to previous data from the year 2000 samples (Figure 4B, and Ref [10]): 290Q, 0.79; 335K, 0.31; 336D, 0.59; 340E, 0.92; 350A, 363S, 1.00; 415G, 0.95; 429E, 0.97; and 440E, 0.97(Figure 5). Notably, 335K was the only position that showed more consistency with the Indian type C origin in the year 2008–2010 data than in the year 2000 data.

### Drug Resistance Mutations in Pro and RT

To research the prevalence of HIV-1 drug resistance, each sequence was submitted to the HIV Drug Resistance Database at Stanford University. All the CRF08_BC sequences from all three cities showed a T69S mutation in their RT region (Table 2), consistent with the results of a recent study of HIV-infected cases in Guangxi Province [12]. This T69S mutation is highly selective in its response to NRTI treatment, yet the effect on NRTI susceptibility has not been well studied. The presence of T69S may provide a certain degree of NRTI resistance, possibly acting together with other mutation(s).

**Table 2:** Drug resistance-associated mutations detected in Guangxi samples.

Mutations	Detected in
PI major resistance	
M46I	BS118 (CRF08_BC)
	NN128 (CRF07_BC)
PI minor resistance	
L10I	BH045 (CRF01_AE)
	NN020 (CRF01_AE)
NRTI resistance	
T69S	All CRF08_BC
K65R	NN137 (CRF01_AE)
NNRTI resistance	
L106I	BH045 (CRF01_AE)
	BH053 (CRF01_AE)
K103R	BH074 (CRF08_BC)
E138A	NN120 (CRF08_BC)

In addition to the T69S mutations, only three CRF08_BC sequences (5.26%) showed additional mutation(s) that produced resistance to anti-HIV drugs (Table 2). A major resistance mutation in PR, M46I, was detected in sample BS118 against protease inhibitor. This same mutation was also detected in NN128, a CRF07_BC strain. Providing resistance to atazanavir, fosamprenavir, indinavir, lopinavir, and nelfinavir, this M46I mutation indicated the occurrence of a low-level transmission of PI-resistant HIV-1 strain(s) in Baise city. K103R and E138A were also found in BH074 and NN120, respectively.

While most drug-resistant mutations were found in the CRF08_BC sequences, an L106I was observed in the RT region of BH045 and BH053, two of the CRF01_AE sequences discovered in Beihai city (Table 2). As is true for K103R (detected in the same region of BH074), the impact of these two mutations was not clear, since L106I is a very common polymorphism for HIV-1 sequences, and K103R appears in 1–2% untreated persons. Also, both of these mutations decrease NNRTI susceptibility only when paired with an additional mutation of V179D, which our sequence did not cover. In addition, a minor PI-resistance mutation, L10I, was found in BH045 and NN020; this mutation is associated with resistance to most PIs when it is present with other mutations, and it can also be detected in 5–10% of untreated patients. While most of the mutations caused only minor drug resistance, an NRTI-resistant mutation K65R was also discovered in NN137, one of the CRF01_AE strains. This mutation introduces intermediate resistance to didanosine, abacavir, lamivudine, emtricitabine, and tenofovir, and it also causes low-level resistance to stavudine and hypersusceptibility to zidovudine.

## Discussion

Different patterns were observed for various HIV-1 subtypes being transmitted in different areas within Guangxi province. Consistent with our previous report [10], a circulating recombinant form between subtypes B and C, CRF08_BC to be exact, was dominant among the IDUs in Baise city (Table 2, and Ref. [6]). Baise is very close to Yunnan province, a location that may have given rise to CRF08_BC. The dominance of CRF08_BC that we observed in Baise confirmed the transmission route of HIV-1 from Yunnan to Guangxi [22], while the substitution of subtype C with CRF08_BC suggested that CRF08_BC offers certain advantage(s) for successful infection.

**Figure 6:**
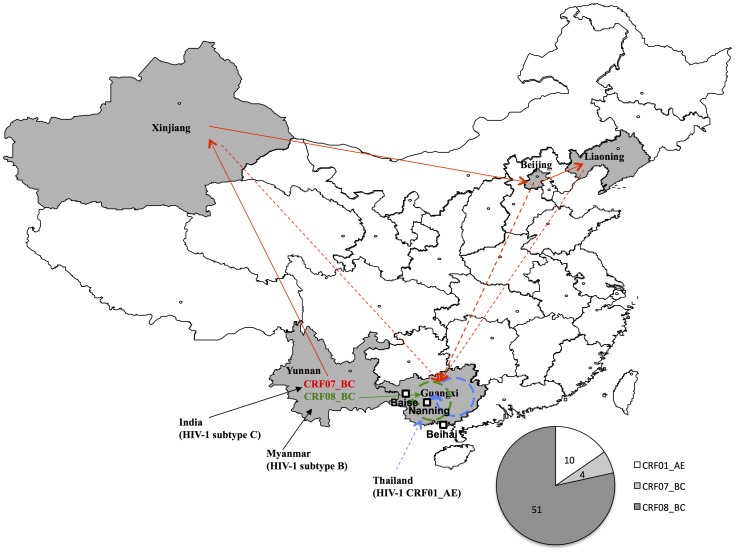
HIV-1 transmission routes related to Guangxi province. Previous reports have suggested that both CRF07_BC and CRF08_BC were generated in Yunnan and transmitted into Xinjiang and Guangxi, respectively through IDU [3], [22]. In the present study, phylogenetic analysis suggested that CRF08_BC may circulate within Guangxi province and that CRF07_BC was introduced from multiple other provinces rather than Yunnan; in contrast, both internal transmission and foreign introduction were detected for CRF01_AE. The provinces involved are labeled in gray, and foreign countries are indicated outside the map of China. Arrows are used to suggest the transmission routes of different HIV subtypes, with blue for CRF01_AE, red for CRF07_BC, and green for CRF08_BC. Routes suggested in this study are indicated by dashed lines. The three cities, Baise, Nanning and Beihai, were located on the map with squares. The pie chart indicated the overall distribution of HIV-1 subtypes retrieved from Guangxi.

As opposed to the relative genetic uniformity of HIV-1 that we observed in Baise city, the degree of genetic diversity was very different in the IDU samples we collected from Nanning and Beihai cities of Guangxi, where multiple subtypes were detected in the phylogenetic tree. However, CRF08_BC was still the main circulating strain. Indeed, over half of the samples were determined to be CRF08_BC, a different result than that reported in a recent study in Liuzhou city [12], which is also located in the eastern area in Guangxi province (but in the north instead of the south). Notably, the second dominant subtype CRF01_AE formed two smaller clusters (with bootstrap values >70) during the phylogenetic analysis (Figure 1). One cluster was formed with a reference sequence collected from Guangxi province, thus suggesting an internal transmission of CRF01_AE strains, while the other one was closer to a sequence from Thailand, indicating possible foreign introduction of CRF01_AE from neighbor countries (Figure 6).

Detection of CRF07_BC in Guangxi was rarely reported; in fact, to our knowledge, the first reported presence of CRF07_BC was in Guilin city by our group in 2002 [18], and later in Liuzhou city by Su et al. [12] in 2011. Our phylogenetic analysis indicated that four CRF07_BC sequences were detected in Nanning and Beihai, but not in Baise. Besides its low-level transmission in Guangxi (4 out of 65 in our study), CRF07_BC in Guangxi possible origininated from northern China (Figure 2 and 6) rather than from its neighbor province Yunnan, where CRF07_BC has been reported to be predominant [8]. This origin resembles that of CRF07_BC in other areas of China [23].

The p6 region of HIV-1 Gag is involved in virus release [24], viral maturation [25], and Pol protein retention [26], [27]. Several P6 deletion patterns have been reported, including 1-, 2-, 3-, 5-, 7-, and 13-aa deletions [19], [28]. All four CRF07_BC sequences in this study showed a 7-aa deletion, including the “KELY” motif, whose function is yet unknown with regard to HIV-1 pathogenesis. Despite the fact that only four sequences were determined to be CRF07_BC, this 7-aa fragment was missing from all these sequences; this finding is not only different from that for previously reported CRF07_BC from Yunnan (and again suggests that Yunnan may not be the origin of CRF07_BC), but it also might indicate that this mutation pattern is under selection pressure and thus has become dominant in at least Beihai and Nanning cities within Guangxi province.

The low-level variation observed in the V3 loop within the Env fragment of both CRF07_BC and CRF08_BC may be determined by local cell tropism, since V3 is one of the most important determinants of viral tropism and co-receptor usage. A similar variation in status was also observed for the C2 region but not for C3, even though both regions are responsible for HIV-1 targeting of CD4 host cells. It has also been shown in a previous study that the Indian subtype C is the origin of the CRF08_BC envelope gene retrieved from Guangxi Province [10]; this finding was consistent with the hypothesized transmission route of HIV-1 [22]. However, our study now suggests a different result for residue positions associated with Indian subtype C, with differences ranging from 0.16 to 0.47. Moreover, in over just 8–10 years, evolution of the residues of the C2-V4 region has apparently produced more variation. This increased variation may not be the result of multiple introductions of HIV-1 trains into Guangxi, as no obvious sub-cluster could be observed in the CRF08_BC cluster (Figure 1). Therefore, our observation may indicate a viral evolution resulting from an adaption by local people during virus transmission over the past few years. Such variations also suggest that future HIV vaccine should induce immune responses that target more conserved regions so that the diversity will not affect vaccine efficacy.

Only a few of the mutations related to the antiviral drug resistance of HIV-1 have been found in our retrieved Gag/Pol sequences; in contrast, one of them, T69S, was detected among all the CRF08_BC sequences from Baise, Beihai, and Nanning. Since none of the patients had been treated with any antiviral drug before providing samples, this NRTI-selective mutation was very likely to have been generated as a result of the NRTI treatment of earlier patient(s), but it might have been retained because of other distinct factors such as the genomic variation of the local people or even environmental conditions (e.g., weather or diet). We did not observe any wild-type T69 in the RT region of any of the 52 CRF08_BC sequences; this result is consistent with similar evidence from a recent study [12] and supports the concept that T69S has been continuously selected during transmission, even among antiviral-naïve IDUs.

Unlike other reports showing various mutations providing resistance against anti-HIV-1 drugs [12], we found very few other mutations related to antiviral resistance. The sequences BH045, BH053, and BH074 could not be confirmed in terms of their antiviral resistance because both L106I and K103R also need V179D in order to demonstrate potency against anti-HIV drugs. However, the PI mutations M46I and L10I were observed in BS118, NN128, and BH45, NN020, respectively, suggesting a low-level transmission of drug-resistant HIV-1 strains in Guangxi Province. The appearance of these PI-resistant mutations was unexpected, since no protease inhibitors are being used in HAART against HIV-1 in China. In addition to natural mutation, the only possible source of such a mutation would be the importation of drug-resistant HIV strains from outside the country. In any case, only one strong drug-resistant strain (NN137) was seen among the 65 sequences examined; therefore, most HIV-1 patients in these cities would mount a good response to antiviral drugs targeting the PR or RT regions.

Guangxi Province has been a member of the HIV Prevention Trial Network (HPTN) for years. It is also the site of an HIV-1 phase II vaccine trial. Our results not only provide the latest data regarding the local distribution of HIV subtypes but also show evidence of ongoing viral evolution in this area. The vaccine trial was conducted based on predominant CRF08_BC, which is still a major circulating subtype in Guangxi (Figure 1). Detection of CRF07_BC raised little concern for the trial, since both subtypes contain a similar subtype-C-originated *env* gene, whose product contributes most to the effectiveness of the vaccine. However, the increasing predominance of CRF01_AE, as well as the variations in CRF08_BC’s envelope protein certainly provide new challenges to the ongoing trial. In fact, the sequences of both the Gag/Pol and Env fragments from all these samples indicate that local HIV-1 strains are evolving, altering amino acids residues that may be critical for antibody recognition. Also, new strains are being introduced from other provinces and other countries. Thus, our study raises an alarm for researchers, indicating that these new features of HIV strains need to be considered in future studies of HIV prevention.
